# Association Between Lipoprotein(a) and Calcific Aortic Valve Disease: A Systematic Review and Meta-Analysis

**DOI:** 10.3389/fcvm.2022.877140

**Published:** 2022-04-25

**Authors:** Qiyu Liu, Yanqiao Yu, Ruixi Xi, Jingen Li, Runmin Lai, Tongxin Wang, Yixuan Fan, Zihao Zhang, Hao Xu, Jianqing Ju

**Affiliations:** ^1^National Clinical Research Center for Chinese Medicine Cardiology, Xiyuan Hospital, China Academy of Chinese Medical Sciences, Beijing, China; ^2^Graduate School, Beijing University of Chinese Medicine, Beijing, China; ^3^Dongzhimen Hospital, Beijing University of Chinese Medicine, Beijing, China

**Keywords:** lipoprotein(a), calcific aortic valve disease, aortic valve stenosis, aortic valve calcification, systematic review and meta-analysis

## Abstract

**Background:**

Preliminary studies indicated that enhanced plasma levels of lipoprotein(a) [lp(a)] might link with the risk of calcific aortic valve disease (CAVD), but the clinical association between them remained inconclusive. This systematic review and meta-analysis were aimed to determine this association.

**Methods:**

We comprehensively searched PubMed, Embase, Web of Science, and Scopus databases for studies reporting the incidence of CAVD and their plasma lp(a) concentrations. Pooled risk ratio (RR) and 95% confidence interval (95% CI) were calculated to evaluate the effect of lp(a) on CAVD using the random-effects model. Subgroup analyses by study types, countries, and the level of adjustment were also conducted. Funnel plots, Egger's test and Begg's test were conducted to evaluate the publication bias.

**Results:**

Eight eligible studies with 52,931 participants were included in this systematic review and meta-analysis. Of these, four were cohort studies and four were case-control studies. Five studies were rated as high quality, three as moderate quality. The pooled results showed that plasma lp(a) levels ≥50 mg/dL were associated with a 1.76-fold increased risk of CAVD (RR, 1.76; 95% CI, 1.47–2.11), but lp(a) levels ≥30 mg/dL were not observed to be significantly related with CAVD (RR, 1.28; 95% CI, 0.98–1.68). We performed subgroup analyses by study type, the RRs of cohort studies revealed lp(a) levels ≥50 mg/dL and lp(a) levels ≥30 mg/dL have positive association with CAVD (RR, 1.70; 95% CI, 1.39–2.07; RR 1.38; 95% CI, 1.19–1.61).

**Conclusion:**

High plasma lp(a) levels (≥50 mg/dL) are significantly associated with increased risk of CAVD.

## Introduction

Calcific aortic valve disease (CAVD) is one of the most common valve disorders (1). CAVD is characterized by calcification and remodeling of the valve leaflets, which often progresses to aortic sclerosis and stenosis, eventually leading to heart failure, angina, death, and other serious adverse cardiovascular events (2, 3). 2020 VHD guideline recommended the intervention of symptomatic AVS mainly apply to SAVR and TAVI. Surgical treatment is not performed routinely in asymptomatic patients (4). With more and more in-depth studies of pathogenesis, researchers are exploring targeted drugs to delay disease progression (5). A meta-analysis included three RCT and five observational studies to analyze the efficacy of ACEI/ARB to CAVD (6), the results showed that there was no statistically significant difference in all-cause mortality between the two groups, but the AVR rate of the treatment group was lower than that of the control group, which needed large-scale RCT to prove. Multiple rigorous RCTs have shown negative efficacy of statins (7–9). Besides, researchers also explored the targets on phosphate/calcium-metabolism and nitric oxide and IGF-1 signaling pathway, which need to be further proved (5).

Globally, about 10–30% of the population has high lp(a) levels ≥50 mg/dl (10). Epidemiological and genetic evidence suggested that high lp(a) concentration had a direct relationship with cardiovascular disease (11). Previous cytological studies have shown that lipoprotein(a) [lp(a)] played an important role in the pathogenesis of CAVD through increasing inflammation and oxidative stress, promoting calcium deposition of valvular interstitial cells (VICs) (12, 13). Mendelian randomization studies suggested that *LPA* genotype, which could mediate the levels of lp(a), had a strong relationship with CAVD (14). Multiple genome-wide association studies had shown that the rs10455872 genetic variant in *LPA*, which was associated with higher lp(a) levels, was independently related to an augmented risk of CAVD (15, 16). However, evidence from clinical studies was inconsistent. Some studies indicated comparing to those without CAVD, patients with CAVD had significantly higher levels of lp(a) (17, 18), while some studies suggested there were no statistical differences in lp(a) levels between CAVD and controls (19). In response to the lack of systematic and comprehensive evidence on the clinical association between lp(a) and CAVD. We performed an extensive systematic review and meta-analysis to assess whether elevated lp(a) significantly affected the incidence of CAVD.

## Methods

The systematic review and meta-analysis were performed following the recommendations of the Preferred Reporting Items for Systematic Reviews and Meta-analyses (PRISMA) (20) and Meta-analyses of Observational Studies in Epidemiology (MOOSE) checklist (21). The protocol has been registered in PROSPERO database (registration number: CRD42021273149).

### Search Strategy

Two authors (Liu QY and Yu YQ) systematically searched the electronic databases, including PubMed (MEDLINE), Embase, Web of Science, and Scopus, up to 31 August 2021. Searching terms included [“lipoprotein(a)” or “lp (a)” or “lipoprotein”] and (“calcific aortic valve disease” or “calcific aortic valve stenosis” or “aortic valve stenosis” or “aortic stenosis” or “aortic valve sclerosis” or “aortic sclerosis” or “aortic valve calcification”) without language or sample size restrictions. The reference lists of relevant reviews, original reports were also searched for potential eligible records. The initial screening of eligible studies was based on the titles and abstracts.

### Study Selection

All cohort studies and case-control studies that had investigated the association between lp(a) and the risk of CAVD were eligible for inclusion. 2016 ESC/EAS Guidelines suggested that lp(a) ≥50 mg/dL had a significant risk of CVD (22). European Atherosclerosis Society Consensus Panel recommended that 50 mg/dL as the cut-off value of lp(a) elevation to screen the risk of CVD (23). In the U.S, the general cut-off value for lp(a) elevation is 30 mg/dL higher (24). Canadian Guidelines for the Management of Dyslipidemia pointed out that lp(a) ≥30 mg/dL continuously increased the risk of CVD (25). Analyzing Chinese studies on the risk of lp(a) cut-off value, the Expert Statement suggested 30 mg/dl might be the cut point for the increased risk of CVD (26). And lots of studies revealed both two cut-off values might be applicable to assess CAVD risk (27, 28). Therefore, we considered 30 and 50 mg/dL as the cut-off points for grouping and merging as most studies reported lp(a) as a categorical variable. The exclusion criteria were as follows: (1) patients with rheumatic diseases; (2) duplications or conference abstracts; (3) missing data and data that were impossible to extract or calculate from the published results. The eligibility of the included studies was assessed by two reviewers (Liu QY and Yu YQ) independently. Any disputes were resolved by consensus with all the authors.

### Data Extraction

Data extraction was completed by two reviewers (Liu QY and Lai RM) independently. If there were disagreements, a third reviewer would be consulted (Ju JQ). Information extracted from each included study comprised first author's name, publication year, country or region where it was performed, study design, and follow-up duration. Demographic data included the number of participants and primary characteristics were obtained.

### Qualitative Assessment

Both cohort studies and case-control studies were estimated the risk of bias according to the Newcastle–Ottawa quality assessment scale (NOS), with a maximum score of 9 (29). The summary scores in 0–3, 4–6, 7–9 scale were classified into high, medium, and low quality. Qualitative assessment was assessed independently by two reviewers (Wang TX and Fan YX). Any disputes were evaluated by the third author (Xi RX).

### Statistical Analysis

The pooled CAVD prevalence was compared between individuals with lp(a) levels <50 vs. ≥50 mg/dL and with lp(a) levels <30 vs. ≥30 mg/dL, respectively. Adjusted risk estimates of CAVD reported directly in the included studies were extracted, including odds ratios (ORs), hazard ratio (HRs), and risk ratios (RRs). Adjusted factors were currently identified risk factors related to the occurrence and development of CAVD, including age, sex, hypertension, smoking, dyslipidemia, BNP, diabetes, and obesity, and at least one of them was required to be adjusted in advance (30). And plasma Lp(a) concentration is primarily genetically determined by variation in the LPA gene (17). Studies without adjusted values were replaced with unadjusted values. Given the incidence of CAVD is <20%, the OR was approximately equal to RR (31, 32). Therefore, RRs and 95% CIs were used to estimate the combined effects.

The overall effect was calculated by a *Z*-test, and a two-tailed *P* < 0.05 was deemed statistically significant. Chi-square Cochran's *Q*-test and I square statistics were used to assess potential heterogeneity among studies. In detail, I square >50% was defined as high statistical heterogeneity, I square of 0–50% indicates low heterogeneity, I square = 0% indicates no heterogeneity, respectively (33). Considering the potential heterogeneity of the included studies, we employed a random-effects model to calculate the pooled RR estimates, and heterogeneity assessment with *P* ≤ 0.10 was considered as a significance set (34).

Subgroup analysis was performed to investigate the source of heterogeneity, based on study types, countries, and the level of adjustment (the number of covariates). Potential publication and small sample bias were evaluated by Egger's and Begg's test (35). The funnel plot was provided for visual inspection of any bias. Statistical analyses were accomplished with Stata (Version 12.0, College Station, Texas).

## Results

### Search Results and Study Characteristics

**Figure 1:**
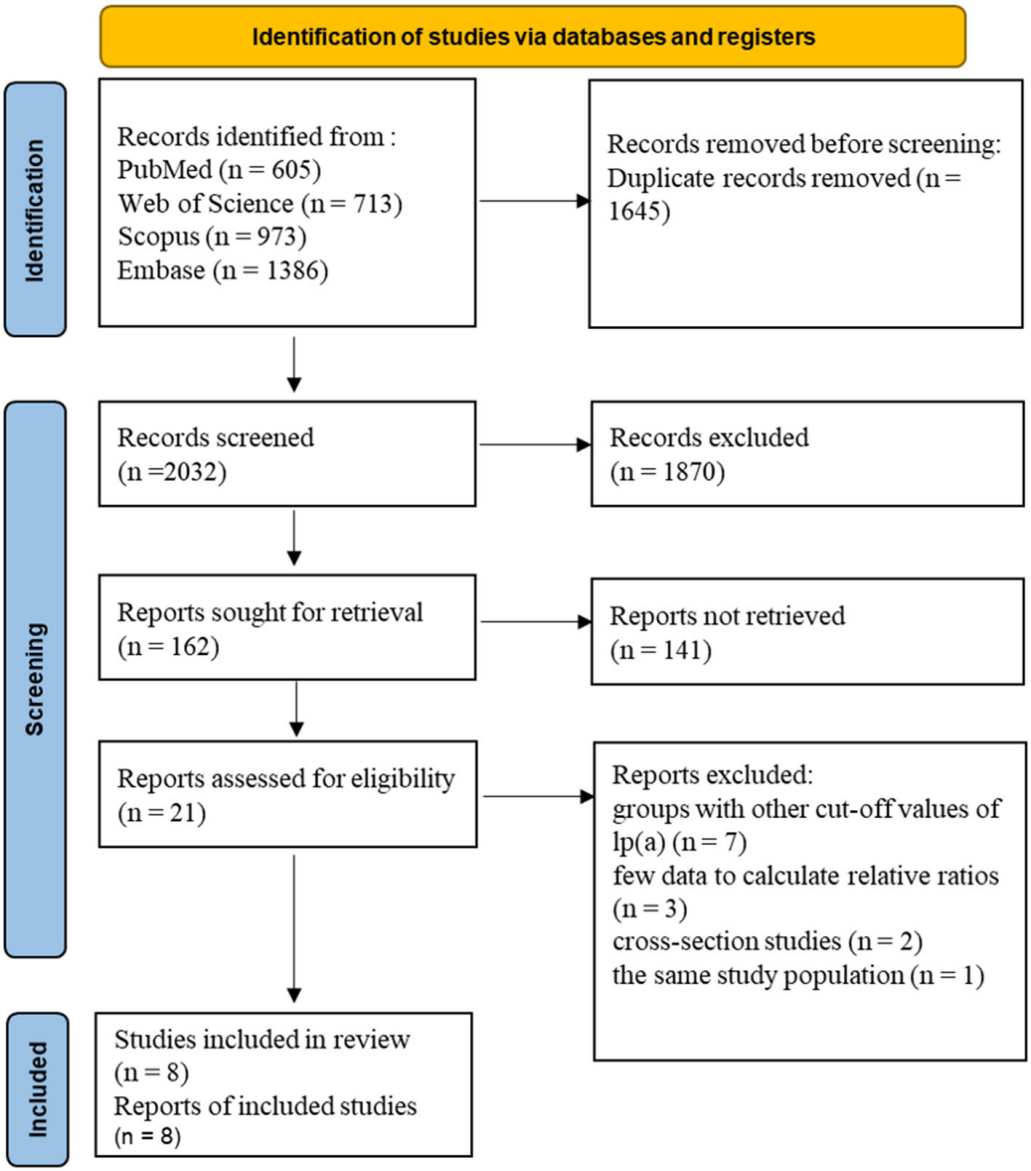
Flow diagram of studies.

A total of 2,032 articles were identified in four databases. After the final full-text screening, eight eligible studies were included for systematic review and meta-analysis (27, 28, 36–41). There were seven studies assessing the association of lp(a) concentration >50 mg/dL with CAVD, five studies assessing the association of lp(a) concentration >30 mg/dL with CAVD. Figure 1 shows a detailed flow diagram.

Of the eight included studies, four were cohort studies, and four were case-control studies. Three included studies originated from America, four from Europe, and the remaining one study was unknown. The age of participants ranged from 51 to 75 years. The proportion of females was 35–57%. Five studies evaluated the association between AVS and lp(a), three estimated associations between aortic valve calcification (AVC) and lp(a). In the four included cohort studies, outcome events occurred in 1,383 of 52,134 participants during follow-up. The case-control studies comprised 425 cases and 372 controls. Characteristics of the included studies are displayed in Table 1. As shown in Table 2, seven studies reported the association using the cut-off value of 50 mg/dL of plasma lp(a), and five studies reported the association using the cut-off value of 30 mg/dL. The adjusted covariates which affected the relationship between lp(a) and CAVD in analyses are provided in Supplementary Table 1. The cardiovascular biomarkers of included studies are shown in Supplementary Table 2.

**Table 1:** The characteristics of included studies.

**Source**	**Country**	**Age, mean**	**Female,%**	**Median of follow-up**	**Participants**	**Events (cases)**	**Specific outcome**	**Diagnosis of CAVD**
**Cohort study**								
Makshood et al. (40)	America	59.3	57	5	695	74	AVC	CT
Afshar et al. (36)	Denmark	58	56	5	29,016	324	AVS	ICD-8,−10 code
Cao et al. (27)	America	61.5	53.7	–	4,678	582	AVC	CT
Zheng et al. (28)	UK	59.2	55.1	19.8	17,745	403	AVS	ICD-10 code
**Case-control study**								
Glader et al. (37)	Sweden	60	40.6	–	202	101	AVS	AVR
Vongpromek et al. (38)	Netherlands	51	37.2	–	129	50	AVC	CT
Nsaibia et al. (41)	NA	71	35	–	300	150	AVS	NA
Wilkinson et al. (39)	America	75	47	–	166	124	AVS	Echocardiography

*CAD, coronary artery disease; AVC, aortic valve calcification; AVS, aortic valve stenosis; AVR, aortic valve replacement; NA, information not available*.

**Table 2:** The statistics of included studies.

**lp(a) 50 mg/dL group**		**lp(a) 30 mg/dL group**	
**Source**	**Risk estimates (95% CI)**	**Source**	**Risk estimates (95% CI)**
Makshood et al. (40)	OR 1.55 (0.71–3.37)	Makshood et al. (40)	OR 1.45 (0.77–2.74)
Afshar et al. (36)	RR 1.95 (1.94–1.97)	Cao et al. (27)	RR 1.38 (1.18–1.62)
Cao et al. (27)	RR 1.44 (1.21–1.72)	Glader et al. (37)	OR 1.7 (0.8–3.9)
Zheng et al. (28)	HR 1.70 (1.33–2.19)	Vongpromek et al. (38)	OR 1.80 (0.88–3.70)
Glader et al. (37)^a^	OR 3.4 (1.1–11.2)	Wilkinson et al. (39)	RR 0.93 (0.78–1.15)
Vongpromek et al. (38)	OR 2.03 (0.80–5.18)		
Nsaibia et al. (41)	OR 4.19 (0.88–19.89)		

*RR, risk ratio; HR, hazard ratio; OR, odds ratio; 95%CI, 95% confidence interval*.

a *This study used 48 mg/dl as the threshold values, we classified it as lp(a) 50 mg/dl group*.

### Quality Assessment

The NOS quality assessment scores ranged from 5 to 8 in cohort studies and 6 to 7 in case-control studies. Two out of four cohort studies were identified as high quality, two as moderate quality. Three out of four case-control studies were identified as high quality, one as moderate quality. Scoring details are provided in Supplementary Table 3.

### Meta-Analysis

Lp(a) 50 mg/dL group analysis We extracted all the effect estimates of CAVD from seven studies grouped with 50 mg/dL of lp(a) level. One study used the Cox regression to calculate the HR. The rest of the studies used logistic regression and reported ORs and RRs.

**Figure 2:**
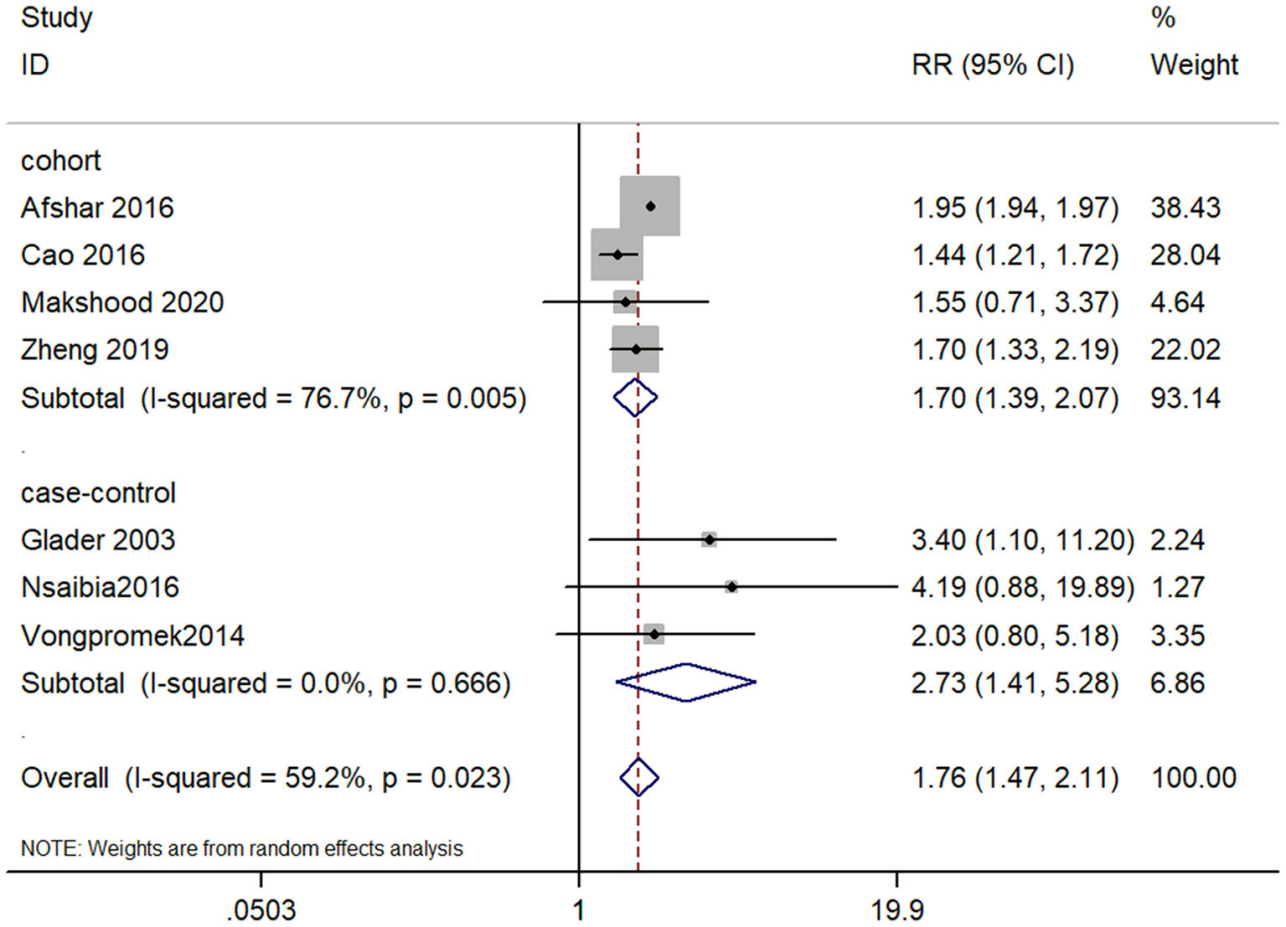
Forest plot for examining the association between lp(a) ≤ 50 mg/dL and CAVD.

Transforming the effect estimates into RRs as described previously, we performed two analyses by study types. The summarized result of the risk ratio was 1.76 (95% CI, 1.47–2.11), which suggested elevated lp(a) level might increase the risk of CAVD by 76% (*P* < 0.001). And lp(a) ≥50 mg/dL might be a risk indicator for CAVD. However, the included studies had substantial statistical heterogeneity (I square = 59.2%), which needed further analysis. The association between lp(a) ≥50 mg/dL and CAVD were significant in both cohort studies (RR, 1.70; 95% CI, 1.39–2.07) and case-control studies (RR, 2.73; 95% CI, 1.41–5.28) (Figure 2).

**Figure 3:**
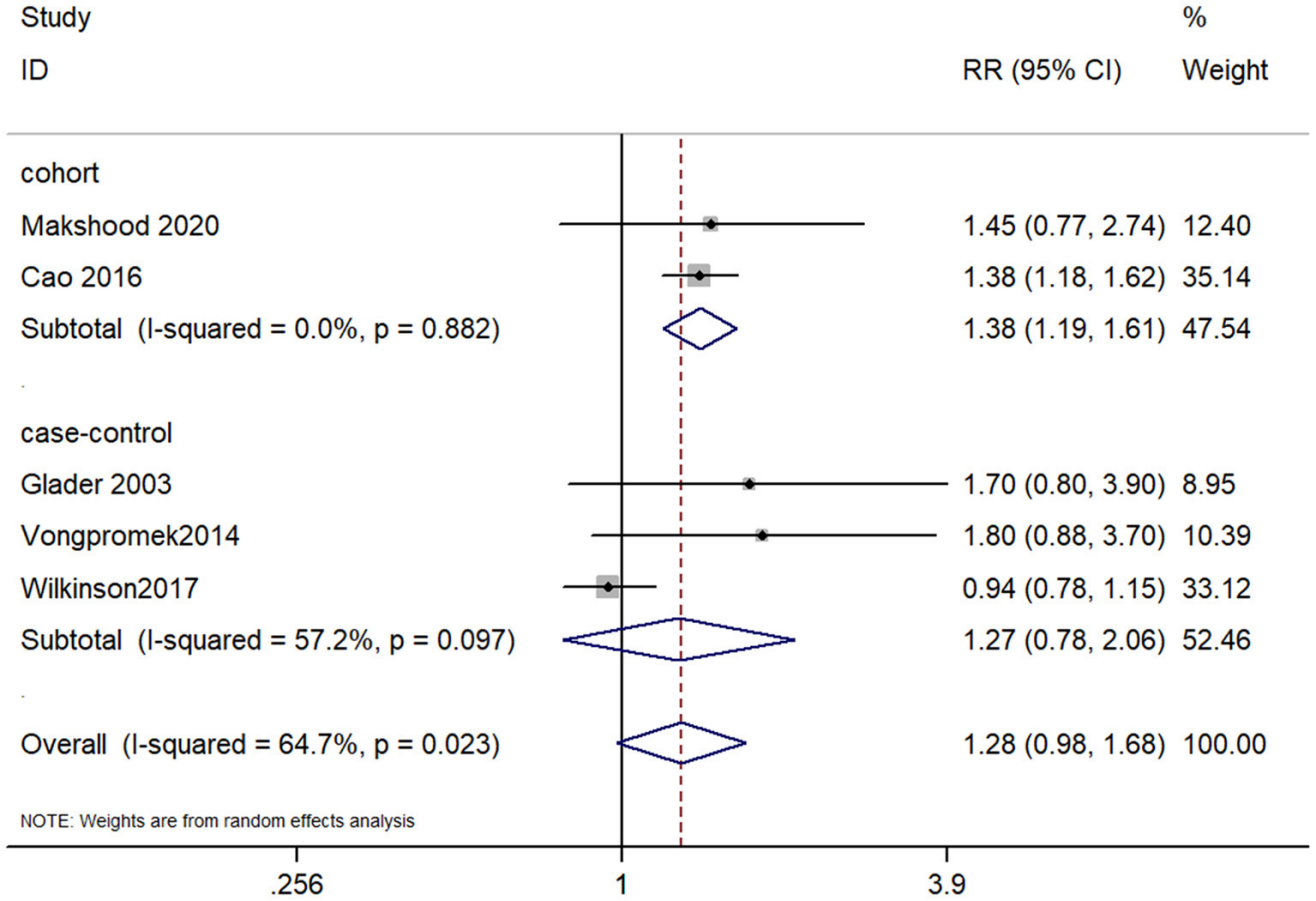
Forest plot for examining the association between lp(a) ≤ 30 mg/dL and CAVD.

Lp(a) 30 mg/dL group analysis Five studies, which consisted of two cohort studies and three case-control studies, examined the association between lp(a) ≥30 mg/dL and CAVD. The pooled RR was 1.28 (95% CI, 0.98–1.68), which indicated lp(a) concentration of 30 mg/dL or higher was not significantly associated with CAVD (*P* = 0.072; Figure 3). There was significant heterogeneity among studies (I square = 64.7%), which needed further analysis. The result of cohort studies showed that lp(a) ≥30 mg/dL might have an association with the increased risk of CAVD (RR, 1.38; 95% CI, 1.19–1.61). Whereas, the case-control studies showed the association between lp(a) and CAVD was not statistically significant (RR, 1.27; 95% CI 0.78–2.06). In summary, the lp(a) cut-off value of 50 mg/dL seemed more convincing than 30 mg/dL as a risk factor for CAVD.

When we screened the relevant studies, many focused on the relationship between lp(a) and AVC or AVS. AVC and AVS are the preclinical and post-clinical phases of CAVD, regarded as the representative of CAVD. So we analyzed the associations of lp(a) with these two outcomes, respectively. In the lp(a) 50 mg/dL group, four studies reported the association with AVS, three reported the association with AVC. The separate meta-analysis indicated that lp(a) ≥50 mg/dL was significantly correlated with AVC (RR, 1.46; 95% CI, 1.23–1.73) and AVS (RR, 1.95; 95% CI, 1.93–1.96). In the lp(a) 30 mg/dL group, two studies reported the association with AVS, three reported the association with AVC. And the relationship of lp(a) ≥30 mg/dL with AVC (RR, 1.40; 95% CI, 1.20–1.63) is stronger than with AVS (RR, 1.11; 95% CI, 0.66–1.87). As shown in Table 3.

**Table 3:** The meta-analyses for the associations of lp(a) with AVC and AVS.

**Outcome**	**RR (95% CI)**	***p*-value**	**I square**
**lp(a) 50 mg/dL group**			
AVC	1.46 (1.23, 1.73)	<0.001	0.0%
AVS	1.95 (1.93, 1.96)	<0.001	0.0%
**lp(a) 30 mg/dL group**			
AVC	1.40 (1.20, 1.63)	<0.001	0.0%
AVS	1.11 (0.66, 1.87)	0.694	50.7%

### Subgroup Analysis

Subgroup analyses stratified according to study types, countries, and level of adjustment, the statistically significant association between lp(a) ≥50 mg/dL and CAVD risk was observed in all subgroups. When the subgroup analysis was conducted considering the study types in lp(a) 30 mg/dL group, a significant positive effect of lp(a) ≥30 mg/dL on CAVD was noted in the cohort studies (RR, 1.38; 95% CI, 1.19–1.61), while the result of case-control studies had a positive trend without statistical significance (RR, 1.27; 95% CI, 0.78–2.06). A subgroup analysis stratified by different countries, lp(a) ≥30 mg/dL was associated with a higher risk of CAVD in Europe (RR, 1.75; 95% CI, 1.03–2.99), but not in America (RR, 1.19; 95% CI, 0.87–1.63). In a subgroup analysis by the level of adjustment (i.e., the median of the adjusted covariates), the pooled RR in studies adjusted for six or more covariates (RR, 1.39; 95% CI, 1.20–1.62) was more marked than in the less than six covariates group (RR, 1.18; 95% CI, 0.64–2.17). The details are shown in Table 4.

**Table 4:** Summary risk estimates of the subgroup analyses.

**Subgroup**	**Design**	**Study (No.)**	**RR (95% CI)**	***p*-value**	**Heterogeneity** **(*I*^2^, *p*-value)**
**lp(a) 50 mg/dL group**					
Study types	Cohort studies Case-control studies	4 3	1.70 (1.39, 2.07) 2.73 (1.41, 5.28)	*p* < 0.001 *p* = 0.003	76.7%, *p* = 0.005 0.0%, *p* = 0.666
Countries	America Europe other	2 4 1	1.45 (1.22, 1.72) 1.95 (1.93, 1.96) 4.19 (0.88–19.89)	*p* < 0.001 *p* < 0.001 –	0.0%, *p* = 0.857 0.0%, *p* = 0.562 –
Level of adjustment	≥7 <7	4 3	1.48 (1.25, 1.75) 1.95 (1.88, 2.02)	*p* < 0.001 *p* < 0.001	0.0%, *p* = 0.521 2.2%, *p* = 0.360
**lp(a) 30 mg/dL group**					
Study types	Cohort studies Case-control studies	2 3	1.38 (1.19, 1.61) 1.27 (0.78, 2.06)	*p* < 0.001 *p* = 0.339	0.0%, *p* = 0.882 57.2%, *p* = 0.097
Country	America Europe	3 2	1.19 (0.87, 1.63) 1.75 (1.03, 2.99)	*p* = 0.282 *p* = 0.038	78.7%, *p* = 0.009 0.0%, *p* = 0.917
Level of adjustment	≥6 <6	3 2	1.39 (1.20, 1.62) 1.18 (0.64, 2.17)	*p* < 0.001 *p* = 0.590	0.0%, *p* = 0.873 65.9%, *p* = 0.087

### Publication Bias

Publication bias assessment of the above two groups was performed using funnel plots at first. There might be asymmetry in the two diagrams figure (Supplementary Figure 1). Therefore, the Egger's test and the Begg's test were carried out for further verification, which suggested these findings might not have publication bias or small sample effect [lp(a)50 mg/dL group: Begg's test, *P* = 0.230; Egger's test, *P* = 0.499; lp(a) 30 mg/dL group: Begg's test, *P* > 0.99; Egger's test, *P* > 0.99].

## Discussion

We evaluated the relationship between elevated plasma lp(a) level and CAVD. The major findings were as follows: (1) The incidence of CAVD was higher in the high lp(a) level group than in the low-level group, as reported by most of the original studies. (2) The pooled results of lp(a) 50 mg/dL group supported that plasma lp(a) ≥50 mg/dL might be a risk factor for CAVD. Whereas, there was insufficient evidence for the association between plasma lp(a) ≥30 mg/dL and CAVD. Also, substantial heterogeneity between studies was not well-explained by subgroup analysis with a 30 mg/dL lp(a) cut-off value, which called for caution when interpreting the result. (3) Patients with plasma lp(a) ≥50 mg/dL might have a higher risk of CAVD than those with lp(a) ≥30 mg/dL. Therefore, we speculated that the magnitude of lp(a) concentration might have a dose-response relationship with CAVD. (4) The progression of CAVD has multiple stages, including calcification and stenosis, etc. AVC, an independent predictor of cardiovascular events (42), not only may progress to AVS (43), but increases the risk of CAD and all-cause mortality (44, 45). Severe AVS directly correlates with heart failure and death (2). Therefore, the lp(a) effect on AVC and AVS were analyzed separately. The results showed that high plasma lp(a) (≥30 mg/dL and ≥50 mg/dL) was associated with AVC, the relationship of AVS with lp(a) ≥50 mg/dL was significant but not with lp(a) ≥30 mg/dL. (5) The subgroup analysis by study types in lp(a) 30 mg/dL group concluded a positive finding in the cohort studies instead of case-control studies. Included case-control studies have the disadvantages of small sample size and the inherent nature of recall and select bias, which might cause inaccurate reporting of the results (46). And due to the larger sample size, the cohort studies had narrower confidence intervals and higher weight with more accurate estimates. Therefore, large sample cohort studies are needed to further explore the relationship between lp(a) ≥30 mg/dL and CAVD. The analysis result in Europe was more significant than in America, and no meaningful association of lp(a) ≥30 mg/dL was found in America. The participants of two American studies mainly involved Asians, Hispanics, Caucasians and Blacks, and the initial results were inconsistent among different races. However, the race of European studies was relatively homogeneous. Therefore, there are possible ethnic effect factors in the pathogenesis of lp(a)-mediated CAVD (27, 40). And a research article from the MESA study pointed out a possible race/ethnicity-related modification of lp(a) and coronary heart disease events (24). Their findings suggested that the 30 mg/dL cutoff for lp(a) is inappropriate in Caucasian and Hispanic individuals, and the higher 50 mg/dL cutoff should be considered. In contrast, the 30 mg/dL cutoff remains suitable in Black individuals. From the present evidence, the cut-off values of lp(a) differed according to different race/ethnicity groups. But most studies agreed on the cut-off values of 30 and 50 mg/dL. That's why we analyzed using both cut-off values in our meta-analysis. Our subgroup analysis results agree with the findings from Guan et al. (24). (6) The Egger's test and the Begg's test had certified that there was no publication bias in these studies. The number of included studies was <10 and the estimates we used were inherently related to their standard errors, leading to relatively imprecise test power of the funnel plot. Whereas, it was undeniable that selection bias and true heterogeneity might also be related to the asymmetry, such as unavailable unpublished studies, which inevitably lead to publication bias. And we conducted the subgroup analysis to investigate the source of heterogeneity, and this deficiency was made up to some extent.

Besides other conventional risk factors like age, sex, hypertension, and smoking, dyslipidemia played a vital role in calcific aortic valve disease (30). Lipids oxidation may promote chronic low-grade inflammation in the aortic valve, which frequently induces the osteogenic process of VICs (47–49). However, whether LDL-C or HDL-C has casual associations with CAVD remains controversial (50, 51). And two meta-analyses about the effect of statins on aortic valve stenosis demonstrated statins might not retard the progression of valve stenosis even though LDL-C concentration was reduced (52, 53). Instead, attention has turned to lp(a). A rapidly-growing body of evidence demonstrated that lp(a) has a bright prospect in predicting and treating CAVD. A previous systematic review speculated plasma lp(a) might be related to the occurrence and progression of AVS. However, this study did not perform a quantitative meta-analysis (54). Our work, which has been thoroughly searched, rigorously screened and quantitatively analyzed, found that lp(a) ≥50 mg/dL could potentially be a proper risk factor for CAVD. Therefore, lp(a) concentration could be more suitable for assessing the incidence of CAVD, compared with that of LDL-C and HDL-C.

Several plausible mechanisms may account for the underlying pathophysiology of lp(a)-mediated CAVD. Lp(a) is a low-density lipoprotein-like structure, which mainly contains apolipoprotein B (apo B) covalently bound to apolipoprotein(a) [apo(a)] (55, 56), transporting some pro-inflammatory and pro-osteogenic mediators. When aortic valve leaflets are damaged to mechanical or shear stress, excessive lp(a) could infiltrate and accumulate the valve to stimulate inflammation, calcification, and fibrosis (57–59). Oxidized phospholipids (OxPL), primarily carried by lp(a) complexes, has been demonstrated the association with CAVD (60, 61). Zheng et al. have revealed that the high content of lp(a) and OxPL-apoB was independently associated with increased active tissue calcification and clinical events such as AVR or all-cause mortality (12). OxPL might be hydrolyzed to lysophosphatidylcholine (LPC) in the presence of phospholipases, promoting valvular inflammation, thickening and mineralization (62). Lipoprotein-associated phospholipase A2 (Lp-PLA2) and autotaxin (ATX) might be the key phospholipases in the metabolism of lp(a)-OxPL. Lp-PLA2, with a high affinity to lp(a), could convert OxPL into LPC, increasing the expression of phosphate-related genes (63, 64). ATX, mainly combined with apo(a) of lp(a) particles, could transform LPC into LysoPA, which drives the inflammatory and osteogenic program through the NF-κB/IL-6/BMP pathway (41, 65). In addition, lp(a) could directly mediate remodeling and calcification of VICs through inducing MAPKs signaling pathway and the expression of pro-osteogenic factors like bone-specific transcription factor SP7 (osterix), BMP-2, and BMP-4 (66).

Welsh et al. revealed reducing the baseline lp(a) levels by 80%, patients with lp(a)≥175 nmol/l and baseline CVD could decrease the risk of CVD by 20% (67). And the conclusions of this study suggested that lowering lp(a) levels might be an important way to intervene CAVD. Pharmacotherapies of lp(a) include PCSK9 inhibitor and nucleic acid antisense, etc. In the FOURIER study, it was found that PCSK9 inhibitors could reduce the plasma Lp(a) level on average 26.9%, and patients with higher Lp(a) levels had a 23% lower risk of major cardiovascular events (68). In the ODYSSEY OUTCOMES trial, the reduction of Lp(a) levels by PCSK9 inhibitors was associated with a decrease in the risk of cardiovascular disease (69). Mipomersen is a 2′-O-methoxyethyl modified second-generation antisense oligonucleotide, which binds to homologous Apo B messenger RNA to inhibit the synthesis of ApoB-100. It can significantly reduce Apo B and Lp(a) levels (70). IONIS-APO(a)Rx, an oligonucleotide targeting lp(a), could reduce lp(a) levels by 66–92%, OxPL-ApoB and OxPL-apo(a) decreased moderately, which play a vital role in pathogenesis of CAVD (71).

There are several strengths of our meta-analysis. Firstly, this is the first meta-analysis assessing the association between lp(a) and CAVD. Secondly, our analysis included a comprehensive search strategy, a considerable number of participants, a subgroup analysis for heterogeneity. And we integrated all relevant cross-sectional and case-control studies, allowing us to determine whether there was a significant relationship between lp(a) and CAVD. Thirdly, most of the included studies have controlled the confounders, which ensured the reliability of the outcomes. Finally, we investigated the relationship between high lp(a) levels (≥50 and ≥30 mg/dL) and the incidence of CAVD, respectively, which provided some guidance for patients with elevated plasma lp(a) levels to assess their risk of CAVD. However, heterogeneity was observed in our meta-analysis, even though most of studies had adjusted multiple potential confounders. Therefore, the results of the present analyses should be interpreted with caution.

## Conclusion

Based on the current cohort studies and case-control studies, we could conclude that patients with lp(a) ≥50 mg/dL were at a significantly high risk of CAVD. However, further large-scale prospective cohort studies with high quality and adequate control for confounders are necessary to draw a firm conclusion on the causality between plasma lp(a) and CAVD.

## Data Availability Statement

The original contributions presented in the study are included in the article/Supplementary Material, further inquiries can be directed to the corresponding author/s.

## Author Contributions

QL and JJ conceived the study. QL and YY searched the studies, screened the studies, analyzed the data, and wrote the manuscript. QL and RL extracted the data. TW, YF, and RX assessed the risk of bias. JL, JJ, and HX reviewed and revised the manuscript. All authors contributed to the article and approved the submitted version.

## Funding

This research was supported by grant CI2021A00917 from the China Academy of Chinese Medical Sciences Innovation Fund, grant ZYYCXTD-C-202007 from the Innovation Team and Talents Cultivation Program of National Administration of Traditional Chinese Medicine, and grant ZZ13-YQ-017 from the Central Public Welfare Research Institutes of China Academy of Chinese Medical Sciences.

## Conflict of Interest

The authors declare that the research was conducted in the absence of any commercial or financial relationships that could be construed as a potential conflict of interest.

## Publisher's Note

All claims expressed in this article are solely those of the authors and do not necessarily represent those of their affiliated organizations, or those of the publisher, the editors and the reviewers. Any product that may be evaluated in this article, or claim that may be made by its manufacturer, is not guaranteed or endorsed by the publisher.
