# Supplementary material for: Association Between Lipoprotein(a) and Calcific Aortic Valve Disease: A Systematic Review and Meta-Analysis
Source: Front Cardiovasc Med. 2022 Apr 25;9:877140. doi: 10.3389/fcvm.2022.877140 (PMC9082602; doi:10.3389/fcvm.2022.877140)
Supplement: Supplementary file 1 [file Data_Sheet_1.docx]

**Supplementary Table 1.** The covariates of included studies

**Supplementary Table 2.** The cardiovascular biomarkers of included studies **Supplementary Table 3**. Quality assessment of included studies

**Supplementary Figure 1**. Publication bias assessment of included studies

**Supplementary Table 1.** The covariates of included studies

| **Source** | **Covariates** |
| --- | --- |
| Makshood 2020 | age, sex, SBP, use of antihypertensive medications, smoking, diabetes, total cholesterol and HDL-C |
| Afshar 2016 | ─ |
| Cao 2016 | age, sex, hypertension, smoking, education status, diabetes, LDL-C, HDL-C, triglycerides, CAC and serum phosphate levels |
| Zheng 2019 | age, sex, LDL‐C,and CAD |
| Glader 2003 | hypertension, BMI, total cholesterol, apo A-I, apo B and smoking |
| Vongpromek2014 | age, BMI, SBP, DBP, duration of statin use, CYS and CAC |
| Nsaibia2016 | CCB, ACE- inhibitors, ARB, statins, clopidogrel, platelet count, LDL-C, left ventricular ejection fraction, CAD, autotaxin, diabetes, hypertension, smoking and HDL-C |
| Wilkinson2017 | ─ |

SBP= systolic blood pressure; HDL-C= high‐density lipoprotein cholesterol; LDL-C= low‐density lipoprotein cholesterol; CAC= coronary artery calcification; CAD=coronary artery disease; BMI= body mass index; DBP= diastolic blood pressure; CYS= cholesterol-year score; CCB= calcium channel blockers; ACE= angiotensin-converting enzyme; ARB= angiotensin receptor blocker.

**Supplementary Table 2.** The cardiovascular biomarkers of included studies

| **Studies** | **Cardiovascular biomarkers** |
| --- | --- |
| Makshood 2020 | age, sex, BMI, SBP, use of antihypertensive medications, smoking, diabetes, total cholesterol, HDL-C, AVC prevalence and aortic valve extent |
| Afshar 2016 | age, sex, total cholesterol, HDL-C, SBP, smoking and diabetes |
| Cao 2016 | age, sex, hypertension, use of antihypertensive medications, smoking, education status, diabetes, use of diabetes medications, LDL-C, HDL-C, triglycerides, AVC presence, AVC severity, CAC and serum phosphate levels |
| Zheng 2019 | age, sex, BMI, LDL‐C, apo A-I, apo B and CAD |
| Glader 2003 | age, sex, SBP, DBP, BMI, total cholesterol, apo A-I, apo B and smoking |
| Vongpromek2014 | age, sex, BMI, SBP, DBP, smoking, total cholesterol, LDL-C, HDL-C, triglycerides, fasting blood glucose, statin medication, duration of statin use, hypertension, bicuspid aortic valve, AVC, CYS and CAC |
| Nsaibia2016 | age, sex, BMI, waist circumference, obesity, peripheral vascular disease, CCB, ACE- inhibitors, ARB, statins, clopidogrel, glucose, platelet count, LDL-C, triglycerides, OxPL-apoB, haemoglobin, creatinine, ATX activity, ATX mass, left ventricular ejection fraction, transvalvular gradient, Aortic valve area, vessel disease, CAD, autotaxin, diabetes, hypertension, smoking and HDL-C |
| Wilkinson2017 | age, sex |

BMI= body mass index; SBP= systolic blood pressure; HDL-C= high‐density lipoprotein cholesterol; AVC= Aortic valve calcium; LDL-C= low‐density lipoprotein cholesterol; CAC= coronary artery calcification; apoA-I= apolipoprotein A-I; apoB= apolipoprotein B; CAD=coronary artery disease; DBP= diastolic blood pressure; CYS= cholesterol-year score; CCB= calcium channel blockers; ACE= angiotensin-converting enzyme; ARB= angiotensin receptor blocker; OxPL-apoB: oxidized phospholipids on apolipoprotein B-100; ATX= autotaxin;

**Supplementary Table 3**. Quality assessment of included studies

| **Source** | **selection** | | | | **comparability** | **exposure** | | | **Total** |
| --- | --- | --- | --- | --- | --- | --- | --- | --- | --- |
|  | 1 | 2 | 3 | 4 | 1 | 1 | 2 | 3 |  |
| Makshood 2020 | ★ | ★ | ★ | ★ | ★★ | ★ | ★ | ─ | 8 |
| Afshar 2016 | ★ | ★ | ★ | ─ | ★ | ★ | ★ | ★ | 7 |
| Cao 2016 | ★ | ★ | ★ | ★ | ★ | ★ | ─ | ─ | 6 |
| Zheng 2019 | ★ | ★ | ★ | ─ | ★ | ─ | ★ | ─ | 5 |
| Glader 2003 | ★ | ★ | ─ | ★ | ★ | ★ | ★ | ★ | 7 |
| Vongpromek2014 | ★ | ─ | ★ | ─ | ★★ | ★ | ★ | ─ | 6 |
| Nsaibia2016 | ─ | ★ | ─ | ★ | ★★ | ★ | ★ | ★ | 7 |
| Wilkinson2017 | ★ | ★ | ★ | ★ | ─ | ★ | ★ | ★ | 7 |


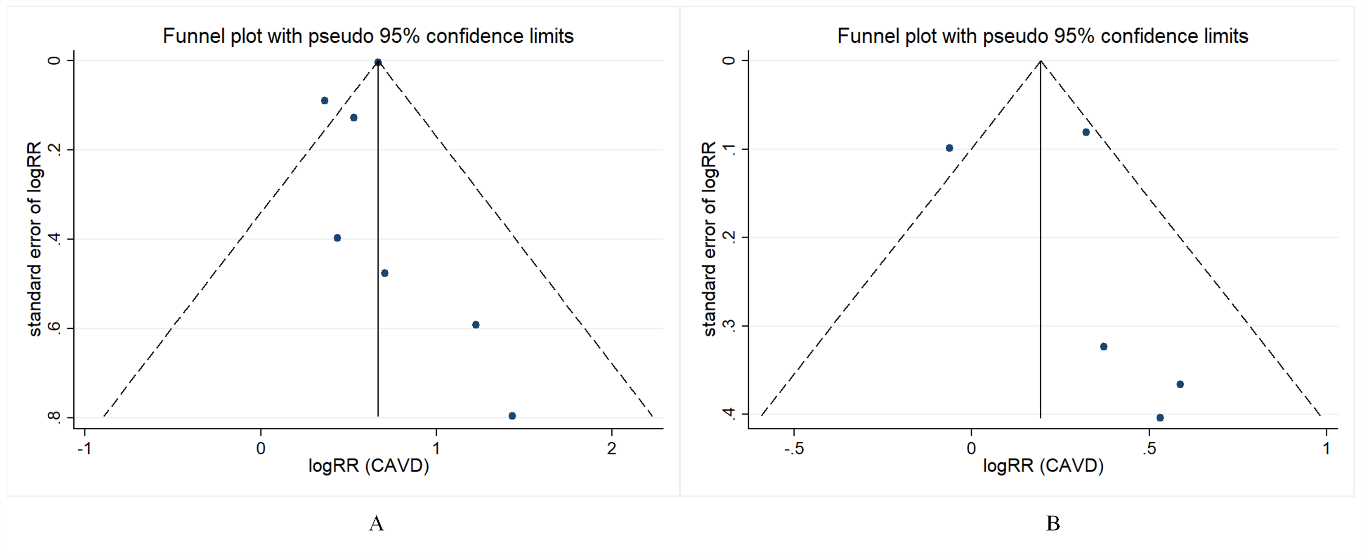


**Supplementary Figure 1**. Publication bias assessment of included studies. (A) lp(a) 50 mg/dL group. (B) lp(a) 30 mg/dL group.
