# Supplementary material for: Association Between Lipoprotein(a) and Calcific Aortic Valve Disease: A Systematic Review and Meta-Analysis
Source: Front Cardiovasc Med. 2022 Apr 25;9:877140. doi: 10.3389/fcvm.2022.877140 (PMC9082602; doi:10.3389/fcvm.2022.877140)
Supplement: Supplementary file 2 [file Presentation_1.PPTX]

## Slide 1
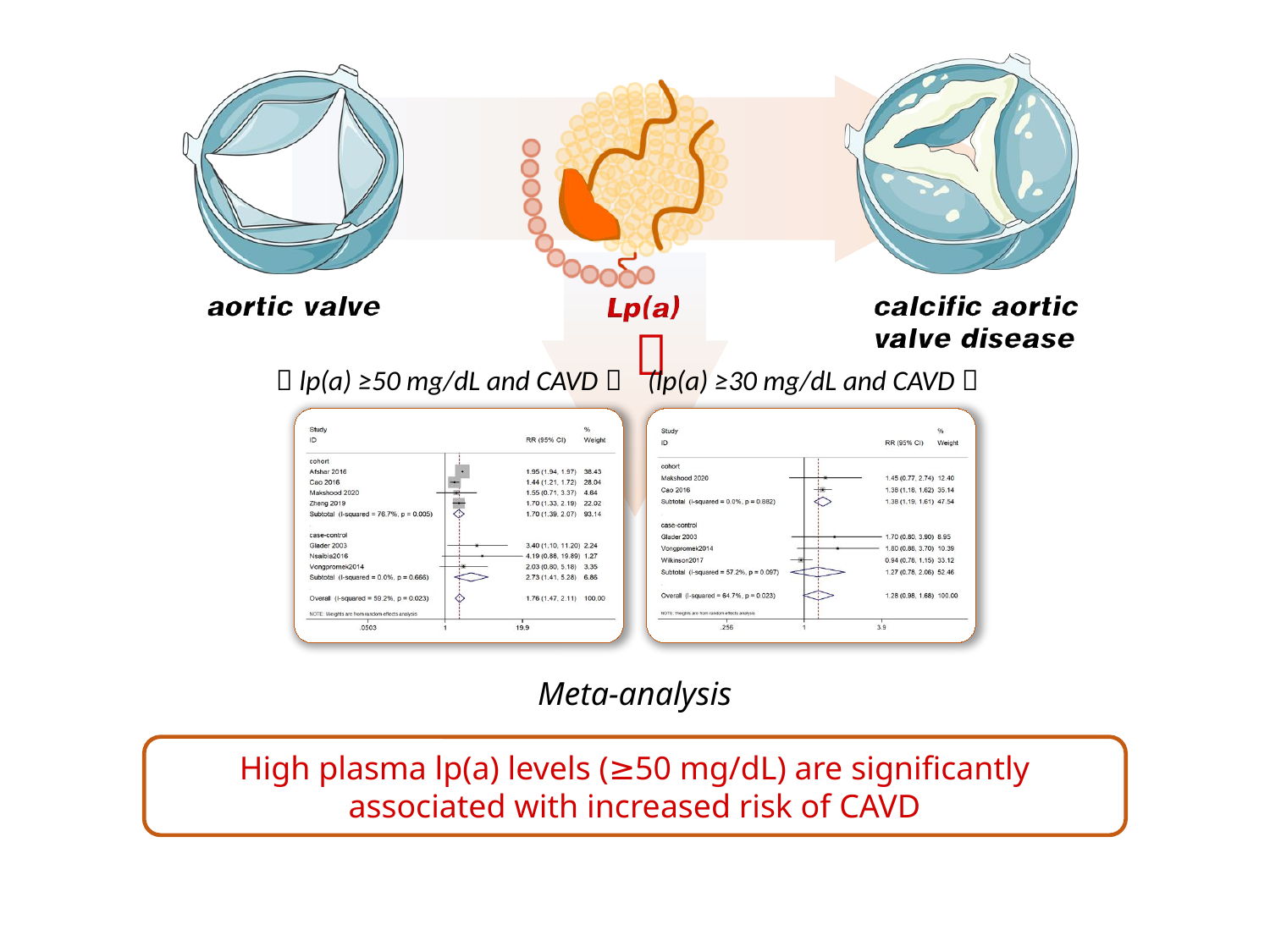

？
（lp(a) ≥50 mg/dL and CAVD）
(lp(a) ≥30 mg/dL and CAVD）
Meta-analysis
High plasma lp(a) levels (≥50 mg/dL) are significantly associated with increased risk of CAVD
